# A Novel Nutrition-Based Nomogram to Predict Prognosis After Curative Resection of Gastric Cancer

**DOI:** 10.3389/fnut.2021.664620

**Published:** 2021-10-25

**Authors:** Hui Zheng, Wenchao Zhu, Zhongfeng Niu, Hongsen Li, Yu Zheng, Zhen Liu, Junlin Yao, Haizhou Lou, Hong Hu, Liu Gong, Hongming Pan, Qin Pan

**Affiliations:** ^1^Department of Medical Oncology, College of Medicine, Sir Run Run Shaw Hospital, Zhejiang University, Hangzhou, China; ^2^Department of Radiology, College of Medicine, Sir Run Run Shaw Hospital, Zhejiang University, Hangzhou, China

**Keywords:** nomogram, gastric cancer, body composition, weight, adjuvant chemotherapy

## Abstract

**Objective:** We sought to investigate the prognostic significance of body composition and weight change during the first 6 months of adjuvant chemotherapy after R0 resection and develop novel nomograms to accurately predict relapse-free survival (RFS) and overall survival (OS).

**Methods:** This retrospective study included 190 patients who underwent curative radical gastrectomy for gastric cancer and received adjuvant chemotherapy. The changes in weight and body composition including skeletal muscle index (SMI), subcutaneous adipose tissue (SAT), and visceral adipose tissue (VAT) were analyzed for 6 months. LASSO Cox regression and multivariate Cox regression were conducted to evaluate other clinical characteristics, which were used to construct a nomogram for the prediction of 3- and 5-year RFS and OS. The constructed nomogram was subjected to 1,000 resamples bootstrap for internal validation. The Concordance index (C-index) and time-dependent receiver operating characteristic (t-ROC) curves were used to evaluate and compare the discriminative abilities of the new nomograms, non-nutritional nomograms, and pTNM stage.

**Results:** The median follow-up duration was 42.0 (25.2–55.1) months. Factors included in the newly-built nomogram for RFS were pT stage, pN stage, tumor site, tumor size, nerve invasion or not, surgery type, and change of L3SMI, while factors included in the nomogram for OS were pT stage, pN stage, tumor size, nerve invasion or not, surgery type, and change of L3SMI. The C-index and t-ROC indicated that our newly-built nomograms had greater potential to accurately predict prognosis than the non-nutritional nomograms and pTNM stage system. Besides, oral nutritional supplements can reduce the degree of weight and L3SMI loss.

**Conclusion:** Change in skeletal muscle mass during adjuvant chemotherapy can be incorporated into predictive prognostic nomograms for RFS and OS in GC patients after radical resection. Dynamic changes in body composition and weight during adjuvant chemotherapy contribute to the early detection of poor outcomes.

## Introduction

According to Global Cancer Statistics 2018, gastric cancer (GC) remains the fifth most commonly diagnosed cancer and the third leading cause of cancer deaths (1). The incidence rates of GC are markedly high in Eastern Asia (e.g., in Japan, Korea, and China) (1, 2). Nearly half of GC patients in China are diagnosed with locally advanced GC, unlike those in Japan and Korea. A previous survey of 1,304 GC patients from more than 100 hospitals in China, and undergoing radical surgery showed that 30 and 55.9% of the patients were stage II and III, respectively, (3) while the corresponding percentage were 13.1 and 12% in Japan, and 12.2 and 10.4% in Korea (4, 5). In the CLASSIC study, capecitabine plus oxaliplatin (XELOX) remarkably improved the 5-year disease-free survival (DFS) compared with surgery alone (6). In the RESOLVE trial, postoperative S-1 combined with oxaliplatin (SOX) was found to be non-inferior to post-operative XELOX for locally advanced GC after D2 gastrectomy (7). The ARTIST and ARTIST II study revealed that the addition of radiotherapy to chemotherapy did not significantly prolong DFS in completely resected GC with D2 lymphadenectomy (8, 9). Thus, patients with stage II-III GC receive adjuvant chemotherapy with a relatively uniform protocol, fluorouracil- and platinum-based regimens (6–10).

The likelihood of disease recurrence in patients with resectable GC is of great significance. Nomograms are considered a more refined method for predicting individualized survival of curatively resected GC. Besides, the nomogram is more predictive than the American Joint Committee on Cancer (AJCC) stage grouping alone. The clinical magnitude of these discrepancies is greatest in stage IIIA, IIIB, and IIIC (11). Relatively common prognostic factors included in a nomogram are age, sex, tumor site, tumor size, depth of invasion, and metastatic lymph nodes (11–15). Kattan et al. (12) developed a nomogram, by including the Lauren classification and the number of negative nodes as prognostic factors. Eom et al. (11) used multi-center data to demonstrate that lymphovascular invasion had a significant prognostic effect on overall survival. Several other clinical factors, including neutrophil-lymphocyte ratio and lymphocyte-monocyte ratio, have been previously incorporated into nomograms (16, 17). More recently the assessment of perioperative body composition and sarcopenia have become clinically useful tools in supporting decision-making in patients with GC (18–20). Due to the influence of many factors, including malabsorption after gastrectomy, side effects induced by adjuvant chemotherapy and postoperative stress response, body weight loss is common in GC patients after surgery, and exacerbated by adjuvant chemotherapy (21). A single-center study by Park et al. (19) reported that patients with a marked loss in at least one body composition parameter had significantly shorter DFS.

In China, the majority of GC patients are found to have locally advanced GC, thus, adjuvant chemotherapy is essential. In the present study, we aimed to delineate the clinical utility of change in weight and body composition assessment in GC patients during adjuvant chemotherapy. A novel nomogram incorporating a nutritional index for predicting long-term outcomes would be highly desirable. Therefore, we also compared the discriminating ability of the newly-built nomogram with one consisting of clinicopathological variables, and pTNM stage.

## Materials and Methods

We retrospectively reviewed the medical records of GC patients who had undergone gastrectomy with D2 lymphadenectomy between January 2013 and December 2018 at Sir Run Run Shaw Hospital. The inclusion criteria were as follows: age ≥ 18 years; curative gastrectomy with at least 15 nodes in the resection specimen; R0 resection; treatment with surgery followed by postoperative chemotherapy; no preoperative treatment or postoperative radiotherapy. Remnant GC and neuroendocrine neoplasm of the stomach were excluded.

The data set included, demographics, operative features, pathological characteristics (including tumor size and differentiation, infiltrating level, number of metastatic, and examined lymph nodes, nerve invasion or not, vascular invasion or not), laboratory tests during perioperative and chemotherapy period, and follow-up data. The TNM stage system in the present study was classified based on the American Joint Committee on Cancer (AJCC, 8th ed., 2018). Adjuvant chemotherapy was administered based on experienced doctors' judgment, and patients' willingness to accept the treatment. The treatment regimen consisted of oxaliplatin plus either S-1(SOX) or capecitabine (XELOX) and the dosage was the same as the clinical trial in the introduction mentioned previously. Any reported toxicity and the use of oral nutritional supplements (ONS) during chemotherapy were documented. There were two types of ONS that our patients are prescribed with. ENSURE® (ABBOTT LABORATORIES B.V.) is a kind of enteral nutritional powder that contains about 450 kcal energy, 15.9 g protein, 15.9 g fat, 60.7 g carbohydrate, as well as vitamins and minerals per 100 g. Ruineng® (Sino-Swed Pharmaceutical Corp. Ltd) is a kind of enteral nutritional emulsion that contains about 650 kcal energy, 29.3 g protein, 36 g fat, 52 g carbohydrate, as well as vitamins and minerals per 500 ml. We retrospectively collected the type and the amount of ONS that our patients are prescribed with in the medical record system during the period of adjuvant chemotherapy. We assumed that every patients took ONS in the amount prescribed by the physician and estimated their daily energy supplement supported by ONS. Besides, chemotherapy dose reductions, delays, or discontinuation for any reason were noted. Relapse-free survival (RFS) was defined as the time from surgery to disease relapse. Overall survival (OS) was defined as the time from surgery to death due to GC.

This study conformed to the Declaration of Helsinki and was approved by the institutional review board of Sir Run Run Shaw Hospital. All patients signed informed consent. A total of 190 patients were included in the study.

## Evaluation of Body Composition

The abdominal CT scans performed within 2 weeks before and 6 months after the initiation of adjuvant chemotherapy were collected as pre-and post-chemotherapy scans, respectively. In every collected abdominal CT scan, the skeletal muscle, visceral adipose tissue (VAT), and subcutaneous adipose tissue (SAT) at the L3 level with both vertebral transverse processes visible were used in the analysis.

The cross-sectional areas of the muscle (cm^2^) at the L3 level computed from each slice were normalized to the square of the height (m^2^) to obtain the L3SMI (cm^2^/m^2^). To assess changes in weight and body composition in different patients during the 6 months of adjuvant chemotherapy, the change between pre-and post-chemotherapy scans was divided by the interval days and multiplied by 180 days.

CT acquisition parameters were as follows: non-enhanced, slice thickness was 5 mm, and the tube voltage was 120 kV. Quantitative measurements were performed by a trained radiologist using Slice O' Matic v 5.0 software (Tomovision, Canada). Established thresholds in Hounsfield units were as follows: skeletal muscle −29 to 150, SAT −190 to −30, and VAT −150 to −50. Boundaries were defined artificially by drawing regions of interest using established cut-off thresholds. Sample image was shown in the Supplementary Materials.

## Statistical Analysis

Statistical analysis was performed using R (version 4.0.3). Descriptive statistics were used to summarize pathological characteristics, changes in weight and body composition, and other characteristics.

The least absolute shrinkage and selection operator (LASSO) method was used to primarily select potential predictive features, solve the collinearity, and avoid over-fitting. Selected predictive factors were further included in the multivariate analysis using a Cox proportional hazards model. Based on the identified predictive factors for RFS and OS in the final model, a nomogram was constructed to predict the 3- and 5-year RFS and OS for GC patients after surgery. The nomogram was internally validated using the bootstrap method with 1,000 resamples. The Concordance index (C-index) ranging between 0.5 and 1.0 and time-dependent receiver operating characteristic (t-ROC) curves were used to evaluate and compare the discriminative abilities of the new-built nomogram, non-nutritional nomogram, and pTNM stage for prediction of RFS and OS. Calibration curves (1,000 bootstrap resamples) for the new nomograms were used to test the consistency between the predicted and actual 3- and 5-year RFS and OS. Improvement in model prognostication was quantified using the net reclassification index (NRI) and integrated discrimination improvement (IDI).

## Results

### Patient Characteristics

The 190 patients with GC included 136 (71.6%) men and 54 (28.4%) women; their median age was 57 years (interquartile range, 52–64). Most of the GC were located in the lower third of the stomach (59.5%). TNM staging was as follows: 10 patients with stage I (5.3%), 34 patients with stage II (17.9%), and 146 patients with stage III 76.8%. A total of 127 (66.8%) and 63 (33.2%) patients were administered with SOX and XELOX regimen, respectively. Seventy-three (38.4%) patients had undergone III or IV degree myelosuppression during adjuvant chemotherapy (neutropenia, leukopenia, thrombocytopenia, or anemia). The weight and body composition (L3SMI, L3VAT, and L3SAT) decreased during chemotherapy as shown in Table 1.

**Table 1:** Patient characteristics.

**Clinicopathological features**	**All (*n* = 190)**
Age (median, IQR)	57 (52–64)
Sex (male/female, *n*)	136/54
Disease characteristics	
Tumor size (cm, median, IQR)	5.2 (3.5–6.0)
Tumor location	
Upper	34 (17.9%)
Middle	43 (22.6%)
Lower	113 (59.5%)
Histologic type	
Differentiated	81 (42.6%)
Undifferentiated	109 (57.4%)
Vascular invasion	
Negative	131 (68.9%)
Positive	59 (31.1%)
Nerve invasion	
Negative	113 (59.5%)
Positive	77 (40.5%)
pT stage	
T1	10 (5.3%)
T2	20 (10.5%)
T3	19 (10.0%)
T4	141 (74.2%)
pN stage	
N0	19 (10.0%)
N1	38 (20.0%)
N2	47 (24.7%)
N3	86 (45.3%)
Surgery	
Distal gastrectomy	108 (56.8%)
Total gastrectomy	82 (43.2%)
CEA (ng/ml)	6.8 (1.4–3.9)
CA199 (U/ml)	49.6 (6.6–22.8)
Adjuvant chemotherapy	
SOX	127(66.8%)
XELOX	63(33.2%)
III or IV degree myelosupression^*^	
No	117 (61.6%)
Yes	73 (38.4%)
Dose reduction or delay	
No	106 (55.8%)
Yes	84 (44.2%)
BMI before chemotherapy (kg/m^2^, median, IQR)	21.2 (19.1–22.8)
Δweight (kg/180 days)	−3.5 (−6.6 to 0.0)
ΔL3SMI (cm^2^/m^2^/180 days)	−4.5 (−8.7 to −0.6)
ΔL3VAT (cm^2^/180 days)	−21.5 (−35.0 to −2.3)
ΔL3SAT (cm^2^/180 days)	−18.6 (−31.3 to −0.7)
ONS during chemotherapy	
No	98 (51.6%)
<500 Kcal/d	59 (31.1%)
≥500 Kcal/d	33(17.4%)

* *myelosupression was graded according to CTCAE version 5.0, IQR interquartile range; CEA carcinoembryonic antigen before surgery, CA 19-9 carbohydrate antigen 19-9 before surgery, BMI body mass index, L3SMI skeletal muscle index at the third lumbar vertebra level; L3VAT visceral adipose tissue at the third lumbar vertebra level; L3SAT subcutaneous adipose tissue at the third lumbar vertebra level; XELOX, capecitabine plus oxaliplatin; SOX, S-1 plus oxaliplatin; ONS oral nutritional supplements*.

### Correlation Between ONS and Body Composition

**Figure 1:**
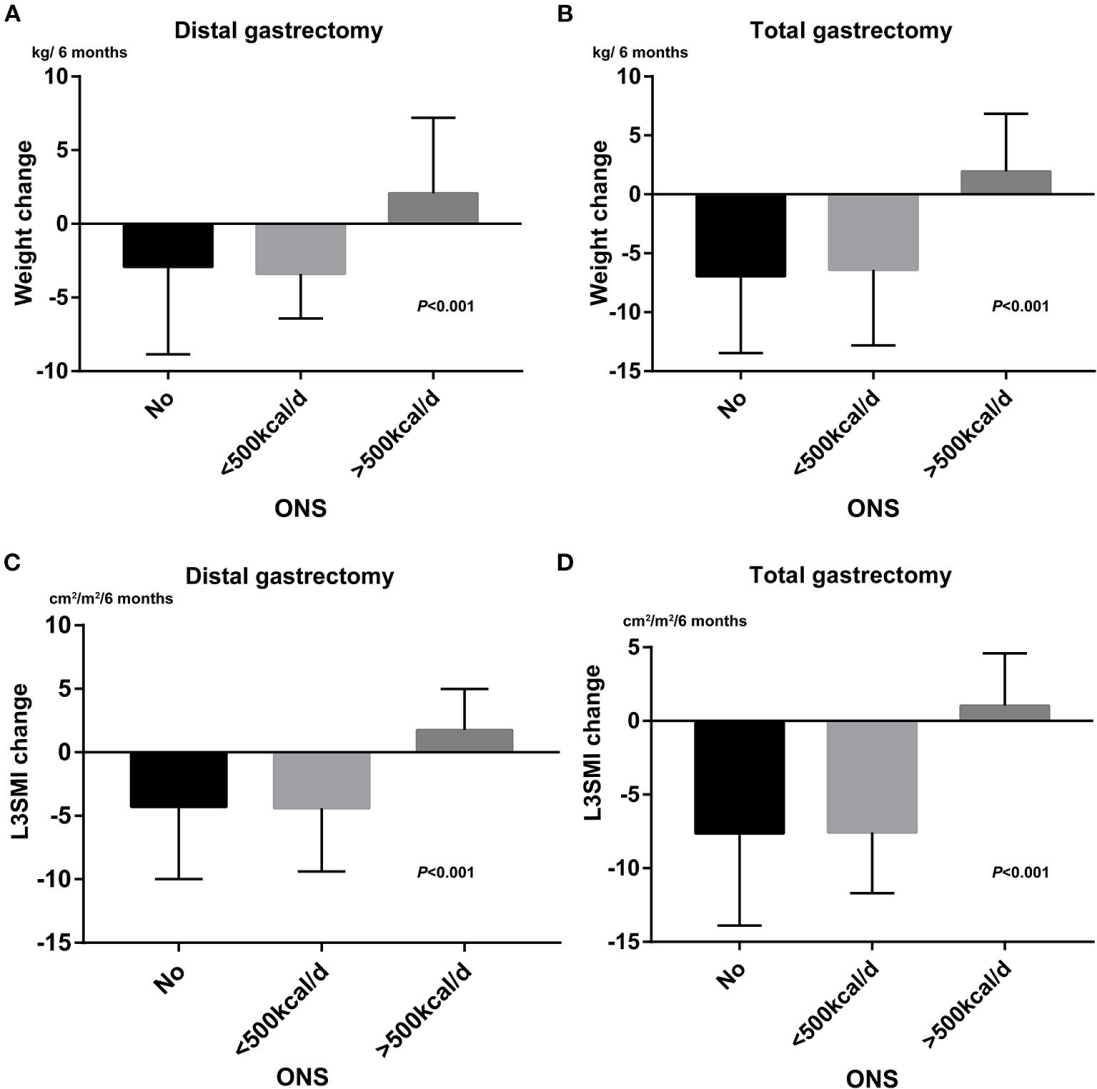
The effects of ONS administration on weight loss and L3SMI loss. **(A)** Bar chart for weight change between different ONS group after distal gastrectomy. **(B)** Bar chart for weight change between different ONS group after total gastrostomy. **(C)** Bar chart for L3SMI change between different ONS group after distal gastrectomy. **(D)** Bar chart for L3SMI change between different ONS group after total gastrectomy.

There were 92 (48.4%) patients who received ONS during the adjuvant chemotherapy period at the doctor's recommendation. Among 92 patients, 33 patients (35.9%) were estimated to take more than 500 Kcal/d from ONS. ONS can reduce the degree of weight and L3SMI loss. The mean value of weight change was −4.4, −5.2, and 2.0 kg/6 months for patients without ONS, patients with <500 Kcal/d, and patients with more than 500 Kcal/d, respectively (*P* < 0.001). The corresponding mean value of L3SMI change was −5.5, −6.3, and 1.5 cm^2^/m^2^/6 months (*P* < 0.001). ONS did not affect L3VAT or L3SAT loss in patients in this study. Weight loss (−5.5 vs. −2.0 kg/6 months *P* < 0.001) and L3SMI loss (−6.4 vs. −3.1 cm^2^/m^2^/6 months *P* < 0.001) were significantly greater after a total gastrectomy than after a distal gastrectomy. As shown in Figure 1, there were significant differences in weight change and L3SMI change between different groups no matter which gastrectomy is followed. Those who were estimated to take more than 500 Kcal/d got more increasement in weight and L3SMI compared to the other two groups.

### Treatment Outcome

The median follow-up duration by December 2020 was 42.0 (25.2–55.1) months. A total of 94 (49.5%) patients had undergone disease recurrence and 61 (32.1%) patients had died from GC during follow-up. Patterns of disease recurrence were distant organ metastasis (*n* = 42, 44.7%), peritoneal metastasis (*n* = 26, 27.7%), distant nodal metastasis (*n* = 20, 21.3%), and anastomotic recurrence (*n* = 6, 6.3%). The 3- and 5-year RFS were 81.1 and 69.4%, while the 3- and 5-year OS were 84.1 and 77.3%, respectively.

Table 2 lists the variables with hazard ratios, which were significantly higher for large tumor size, GC located on the upper part of the stomach, undifferentiated type, advanced pT stage and pN stage, vascular invasion, nerve invasion, total gastrectomy, and loss of weight, L3SMI, and L3SAT during adjuvant chemotherapy. However, age, sex, chemotherapy regimen, and a dose reduction or delay were not significantly associated with prognosis.

**Table 2:** Univariate Cox analysis for replase-free survival and overall survival stratified by clinical factors.

**Factors**	**RFS**		**OS**	
	**HR, 95% CI (days)**	***P***	**HR, 95% CI (days)**	***P***
Gender		0.358		0.411
Male	1 [Reference]		1 [Reference]	
Female	1.226 (0.794–1.894)		1.252 (0.732–2.140)	
Age		0.677		0.412
<65	1 [Reference]		1 [Reference]	
≥65	0.899 (0.543–1.487)		0.761 (0.396–1.462)	
Tumor size	1.123 (1.056–1.194)	<0.001	1.146 (1.070–1.228)	<0.001
Tumor location				
Upper	1 [Reference]		1 [Reference]	
Middle	0.551 (0.310–0.978)	0.042	0.792 (0.403–1.554)	0.497
Lower	0.415 (0.254–0.677)	<0.001	0.389 (0.209–0.726)	0.003
Histologic type		0.005		0.047
Undifferentiated	1 [Reference]		1 [Reference]	
Differentiated	0.532 (0.343–0.825)		0.576 (0.335–0.992)	
Vascular invasion		0.001		0.010
Positive	1 [Reference]		1 [Reference]	
Negative	0.514 (0.340–0.775)		0.512 (0.308–0.851)	
Nerve invasion		0.001		0.001
Positive	1 [Reference]		1 [Reference]	
Negative	0.492 (0.327–0.740)		0.413 (0.247–0.689)	
pT stage	1.969 (1.359–2.852)	<0.001	2.298 (1.337–3.950)	0.003
pN stage	1.673 (1.320–2.121)	<0.001	1.696 (1.256–2.290)	0.001
Surgery		<0.001		<0.001
Total gastrectomy	1 [Reference]		1 [Reference]	
Distal gastrectomy	0.451 (0.299–0.681)		0.310 (0.182–0.529)	
CEA (ng/ml)	0.996 (0.984–1.008)	0.489	0.998 (0.988–1.008)	0.745
CA199 (U/ml)	1.001 (1.000–1.001)	0.198	1.000 (0.999–1.001)	0.667
Chemotherapy		0.712		1.000
XELOX	1 [Reference]		1 [Reference]	
SOX	1.104 (0.653–1.868)		1.000 (0.520–1.922)	
III or IV degree myelosupression^*^		0.698		0.015
No	1 [Reference]		1 [Reference]	
Yes	1.086 (0.716–1.647)		1.869 (1.130–3.091)	
Dose reduction or delay		0.799		0.420
Yes	1 [Reference]		1 [Reference]	
No	0.949 (0.631–1.426)		0.812 (0.491–1.345)	
Δweight (kg/180 days)	0.967 (0.938–0.997)	0.030	0.972 (0.936–1.010)	0.148
ΔL3SMI (cm^2^/m^2^/180 days)	0.913 (0.882–0.945)	<0.001	0.898 (0.859–0.939)	<0.001
ΔL3VAT (cm^2^/180 days)	1.003 (0.998–1.009)	0.246	1.003 (0.997–1.010)	0.317
ΔL3SAT (cm^2^/180 days)	0.990 (0.983–0.996)	0.001	0.991 (0.983–0.999)	0.034
ONS during chemotherapy		0.018		0.052
No	1 [Reference]		1 [Reference]	
<500 Kcal/d	1.154 (0.747–1.783)		1.656 (0.966–2.838)	
≥500 Kcal/d	0.380 (0.181–0.801)		0.663 (0.291–s1.515)	

* *myelosupression was graded according to CTCAE version 5.0; CEA carcinoembryonic antigen before surgery, CA 19-9 carbohydrate antigen 19-9 before surgery, L3SMI skeletal muscle index at the third lumbar vertebra level; L3VAT visceral adipose tissue at the third lumbar vertebra level; L3SAT subcutaneous adipose tissue at the third lumbar vertebra level; XELOX, capecitabine plus oxaliplatin; SOX, S-1 plus oxaliplatin*.

### Construction of the Prognostic Nomograms for RFS and OS

**Figure 2:**
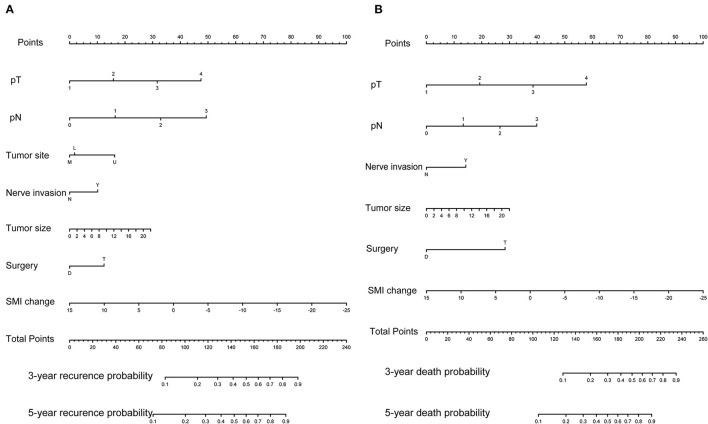
Predictive nomograms. **(A)** Nomogram for predicting 3- and 5-year probability of disease recurrence. **(B)** Nomogram for predicting 3 and 5 month death probability.

Initially, 13 variables including pT stage, pN stage, tumor site, tumor size, tumor differentiation, nerve invasion or not, vascular invasion or not, surgery type, change of weight, L3SMI, L3VAT, and L3SAT, and ONS were included in the analysis. Based on the results of LASSO Cox regression analysis, pT stage, pN stage, tumor site, tumor size, nerve invasion or not, surgery type, change of L3SMI were screened out for RFS, while pT stage, pN stage, tumor size, nerve invasion or not, surgery type, change of L3SMI were screened out for OS. For better outcome prediction, nomograms integrating the selected prognostic factors (7 factors for RFS and 6 factors for OS) were constructed (Figure 2). The nomograms were used by summing the points identified on the points scale for each variable. The added score projected on the bottom scale indicated the probability of 3- and 5-year RFS and OS. Table 3 lists the selected variables with the hazard ratios.

**Table 3:** Selected variables according to the cox proportional hazards regression model.

**Factors**	**RFS**		**OS**	
	**HR (95% CI)**	***P***	**HR (95% CI)**	***P***
pT stage	1.53 (1.04–2.25)	0.03	1.75 (0.99–3.08)	0.053
pN stage	1.55 (1.21–1.99)	<0.001	1.47 (1.08–2.01)	0.015
Nerve invasion				
Negative	1 [Reference]		1 [Reference]	
Positive	1.32 (0.86–2.01)	0.212	1.52 (0.89–2.58)	0.129
Surgery				
Distal gastrectomy	1 [Reference]		1 [Reference]	
Total gastrectomy	1.40 (0.76–2.54)	0.278	2.28 (1.32–3.95)	0.003
Tumor size	1.04 (0.97–1.11)	0.311	–	–
Tumor site				
Lower	1 [Reference]		–	
Middle	0.95 (0.51–1.80)	0.883	–	–
Upper	1.47 (0.75–2.90)	0.260	–	–
ΔL3SMI per 180 days	0.94 (0.90–0.97)	<0.001	0.93 (0.89–0.98)	0.003

*L3SMI skeletal muscle index at the third lumbar vertebra level*.

### Validation and Comparison of the Prognostic Model

**Figure 3:**
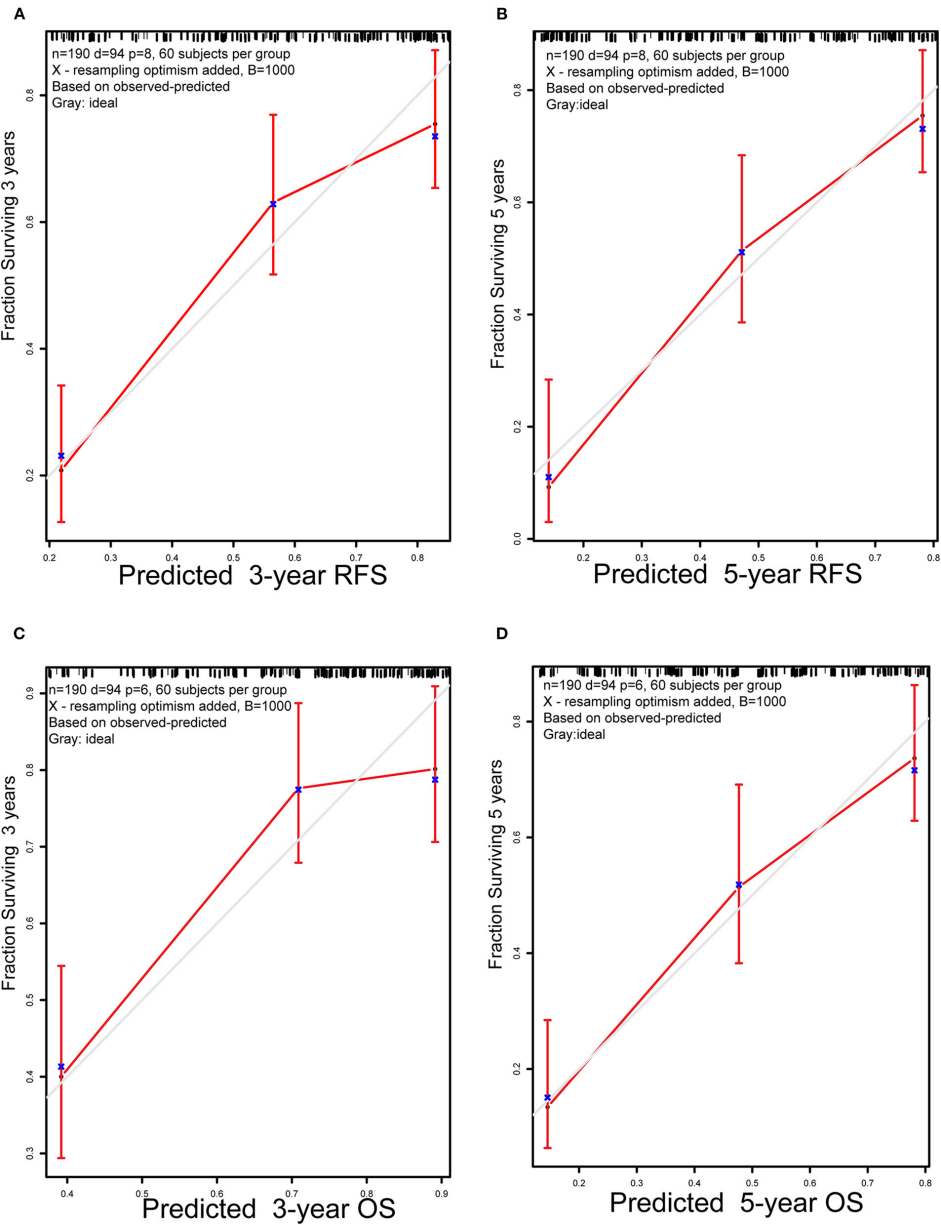
Calibration plots. **(A)** 3-year and **(B)** 5-year RFS nomogram calibration plots; **(C)** 3-year and **(D)** 5-year OS nomogram calibration plots; RFS, replase-free survival; OS: overall survival.

**Figure 4:**
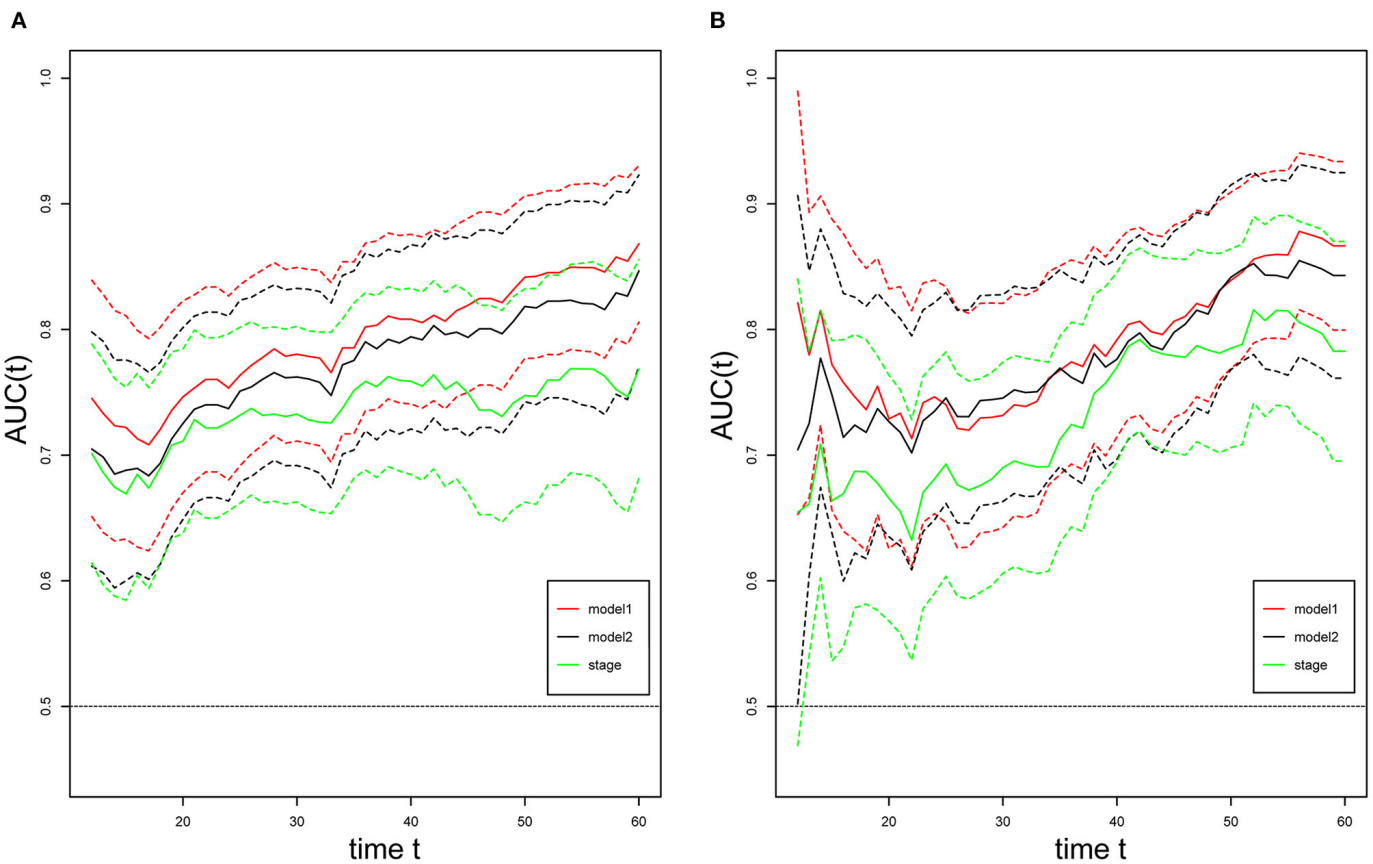
t-ROC curves. Time-dependent receiver-operating characteristic (ROC) curves for the newly-built nomogram, non-ΔL3SMI nomogram, and pTNM for the prediction of replase-free survival **(A)** and overall survival **(B)**. The horizontal axis represents month after surgery, and the vertical axis represents the estimated area under the ROC curve for survival at the time of interest. Red, green, and black solid lines represent the estimated AUCs of the newly-built nomogram (model1), non-ΔL3SMI nomogram (model2), and pTNM, respectively, and broken lines represent the 95% confidence intervals of each AUC. AUC, area under the curve; pTNM, pathologic TNM.

For internal validation, the calibration curves indicated excellent agreement between the predicted and actual survival outcomes of 3- and 5-year RFS and OS (Figure 3). To demonstrate the significance of the newly-built nomograms, we generated t-ROC curves (Figure 4) and used them to compare the prognostic accuracy of the three prognostic models, including nomograms based on ΔL3SMI, nomograms without ΔL3SMI, and pTNM stage and listed the C-index values of the 3- and 5-year RFS and OS as shown in Table 4. The accuracy of the nomograms based on ΔL3SMI was consistently superior to that of non- ΔL3SMI nomograms, and of the pTNM stage throughout the follow-up period. The IDI for the 3- and 5-year RFS was 0.048 (95%CI: 0.000–0.090) and 0.064 (95%CI: 0.008–0.117), while for the 3- and 5-year OS was 0.041 (95%CI: −0.016 to 0.107) and 0.063 (95%CI: −0.030 to 0.133), respectively. The NRI for the 3- and 5-year RFS was 0.196 (95%CI: −0.085 to 0.431) and 0.355 (95%CI: −0.072 to 0.617), and for the 3- and 5-year OS was 0.148 (95%CI: −0.129 to 0.436) and 0.157 (95%CI: −0.165 to 0.536), respectively. These results indicated that the newly-built nomograms had greater potential to accurately predict prognosis compared to the nomograms without ΔL3SMI, especially for RFS.

**Table 4:** The C-index values of different models for 3- and 5-year RFS and OS.

**Models**	**RFS**	**OS**
	**C-index (95% CI)**	**C-index (95% CI)**
Nomogram based on ΔL3SMI		
3-year	0.802 (0.735–0.869)	0.788 (0.708–0.869)
5-year	0.868 (0.806–0.931)	0.871 (0.801–0.940)
Nomogram without ΔL3SMI		
3-year	0.790 (0.720–0.861)	0.783 (0.705–0.862)
5-year	0.847 (0.770–0.923)	0.854 (0.705–0.862)
pTNM		
3-year	0.759 (0.688–0.830)	0.723 (0.639–0.807)
5-year	0.769 (0.682–0.856)	0.781 (0.688–0.873)

*L3SMI: skeletal muscle index at the third lumbar vertebra level*.

## Discussion

The present study focused on the construction of a nomogram for prognosis prediction in GC patients treated with adjuvant chemotherapy after surgery. The clinical variables included, pT stage, pN stage, tumor site, tumor size, nerve invasion or not, surgery type, change in L3SMI for RFS and pT stage, pN stage, tumor size, nerve invasion or not, surgery type, and change of L3SMI for OS. Here, for the first time, we incorporated a dynamic change of skeletal muscle during adjuvant chemotherapy into the nomograms for GC after R0 resection. The predicting power of the newly-built nomograms was compared with the nomograms without ΔL3SMI and pTNM stage system and concluded that the newly-built nomogram was superior.

The primary cause of GC-related death is recurrence. Feng et al. (22) found that the first recurrence peak nearly occurred 3 years after surgery and rose to a maximum at 1.5 years after surgery. Since the length of chemotherapy has been suggested to not exceed 1 year, it is important to clarify the risk factors of recurrence and help doctors advise close follow-up and develop a further treatment plan. The 8th edition of the pTNM stage system provides a useful tool for precision treatment for GC, and the sub-classifying of stage III has been adjusted (23). Accurate prediction of prognosis necessitates enough regional lymph nodes examined during radical surgery. Some researchers have incorporated the ratio of positive lymph nodes or the number of harvested lymph nodes into prognostic nomograms (13, 15). In our study, patients were excluded, if the number of harvested lymph nodes was <15, and the mean value of harvested lymph nodes was 35.3 (24.3–44.0). Considering that the lymph node dissection has become more thorough in recent years, the number of harvested lymph nodes was not included in our final model. Similar to previous studies, tumor location was classified as an upper third, middle third, and lower third. The proportion of upper third GC in our study was relatively low, which is comparable to Han's nomogram (12.4 vs. 17.9%) (13). Consistent with previous findings, the upper third GC is recognized as an indicator of poor prognosis and projected a higher score in our nomogram for RFS. Nevertheless, the differences in prognosis between the middle and upper third tumor locations remain controversial based on this study's findings and those from previous studies (24, 25).

Marked loss of muscle, as an independent prognosticator of compliance with treatment and survival outcomes in GC, has been well established (14, 20, 26–28). This study evaluated change in body composition during adjuvant chemotherapy based on the L3 level calculated by CT images. Univariate Cox analysis revealed that the loss of weight, L3SMI, and L3SAT were all significantly associated with shorter RFS and OS. Weight loss > 5% over the past 6 months is widely regarded as a sign of entering the cachexia period. In our study, weight loss failed to be included in the nomograms by LASSO Cox regression analysis, whereas skeletal muscle change was identified as a prognostic factor in the final model. This result shows that loss of skeletal muscle may be a more accurate indicator of deterioration of nutritional status than the loss of weight. There is a clear shift in the definition of cancer cachexia that requires oncologists assess muscle loss, rather than simply weight loss (29). Sophie et al. found that SMI trajectory, but not a BMI loss, was significantly associated with disease progression (30). Muscle mass loss, irrespective of weight loss, may serve as a sensitive criteria for the early selection of the pre-cachexia age. The reasonable explanation of this phenomenon is that weight loss may be masked by fat gain, additional water in the form of edema, ascites, or pleural effusion.

Adipose tissue is an energy reserve and loss of adipose tissue is also an important part of nutritional deficiency. Unlike skeletal muscle, to date, there has been little agreement about the precise role of visceral and subcutaneous adipose tissue in predicting survival. Some researchers found that increased visceral fat independently predicts surgical complications or high visceral fat is associated with shorter OS (31, 32). However, other researchers reported that a marked loss of visceral fat predicted poor survival (19, 28). In this study, we failed to reveal the prognostic significance of the loss of visceral fat, though stratified by the BMI of subjects, and more high powered studies are needed. Black et al. reported that reduced SAT was associated with poor survival for patients with colorectal cancer and this phenomenon was not found in esophagogastric cancer (33). Dong et al. (34) concluded that high SAT did not significantly influence survival in overweight patients, but was associated with better survival in non-overweight patients. Most patients (90%) were non-overweight in this study, and loss of L3SAT was a negative predictor.

ONS has gained wide-spread acceptance as a nutritional support therapy. Since malnutrition is associated with negative outcomes, the use of proper nutritional support therapy is highly desirable for improved prognosis in GC patients. Previous studies demonstrated that post-discharge ONS in GC patients after surgery improved skeletal muscle maintenance (35) and diminish postoperative weight loss (36). In our results, those who were estimated to take <500 Kcal/d from ONS did not show any improvement in weight and L3SMI change. ONS would only show positive effects in improving the nutritional status when the intake was more than 500 Kcal/d. More professional guidance and regular follow up need to be recommended for GC patients after gastrectomy.

There were several limitations to our study. First, it is a retrospective single-center study, with a small sample size, which limits the generalization of the results. Although 1,000 bootstrap re-samplings were performed to validate this model, external validation using cohorts from other centers was unavailable in our study. Despite the small sample size, alterations in the effects of body composition on survival were striking when other well-established prognostic factors were taken into account. Second, the daily energy supplement from ONS was based on estimates that were subject to error. Third, we evaluated body composition and weight at only two-time points (pre-chemotherapy and 6 months after initiation of chemotherapy). Thus, the impact of the changes in body composition and weight at 3 months or 1 year is unknown. Finally, compliance with ONS, total calorie intake, muscle strength, and physical activity was not evaluated in this study. Therefore, multicenter prospective randomized clinical trials with large sample sizes are needed to confirm these results.

In conclusion, change in skeletal muscle during adjuvant chemotherapy can be incorporated into prognostic nomograms for RFS and OS in GC patients after radical surgery. In patients with severe loss of L3SMI during adjuvant chemotherapy, the decision for subsequent follow-up should be made after deliberate consideration.

## Data Availability Statement

The raw data supporting the conclusions of this article will be made available by the authors, without undue reservation.

## Ethics Statement

Written informed consent was obtained from the individual(s) for the publication of any potentially identifiable images or data included in this article.

## Author Contributions

QP and HZ: conceptualization. HLi, WZ, and ZN: data collection. HZ and HLi: formal analysis. YZ and HP: funding acquisition and project administration. QP and HLo: investigation methodology. WZ and ZN: resource and software. HH and LG: supervision, validation, and visualization. HZ: roles/writing—original draft. QP: writing—review and editing. All authors contributed to the article and approved the submitted version.

## Funding

This work was supported by Zhejiang medical and health science and technology program (No. 2018ZD029) and Zhejiang Provincial Natural Science Foundation of China under grant no. LSY19H160006.

## Conflict of Interest

The authors declare that the research was conducted in the absence of any commercial or financial relationships that could be construed as a potential conflict of interest.

## Publisher's Note

All claims expressed in this article are solely those of the authors and do not necessarily represent those of their affiliated organizations, or those of the publisher, the editors and the reviewers. Any product that may be evaluated in this article, or claim that may be made by its manufacturer, is not guaranteed or endorsed by the publisher.

## Supplementary Material

Supplementary Figure 1 The body composition analysis were segmented using standard Hounsfield unit (HU) and different color (skeletal muscle −29 to 150: red, SAT −190 to −30: yellow, and VAT −150 to −50: blue).
