# Supplementary figures and images for: A Novel Nutrition-Based Nomogram to Predict Prognosis After Curative Resection of Gastric Cancer
Source: Front Nutr. 2021 Oct 25;8:664620. doi: 10.3389/fnut.2021.664620 (PMC8572887; doi:10.3389/fnut.2021.664620)

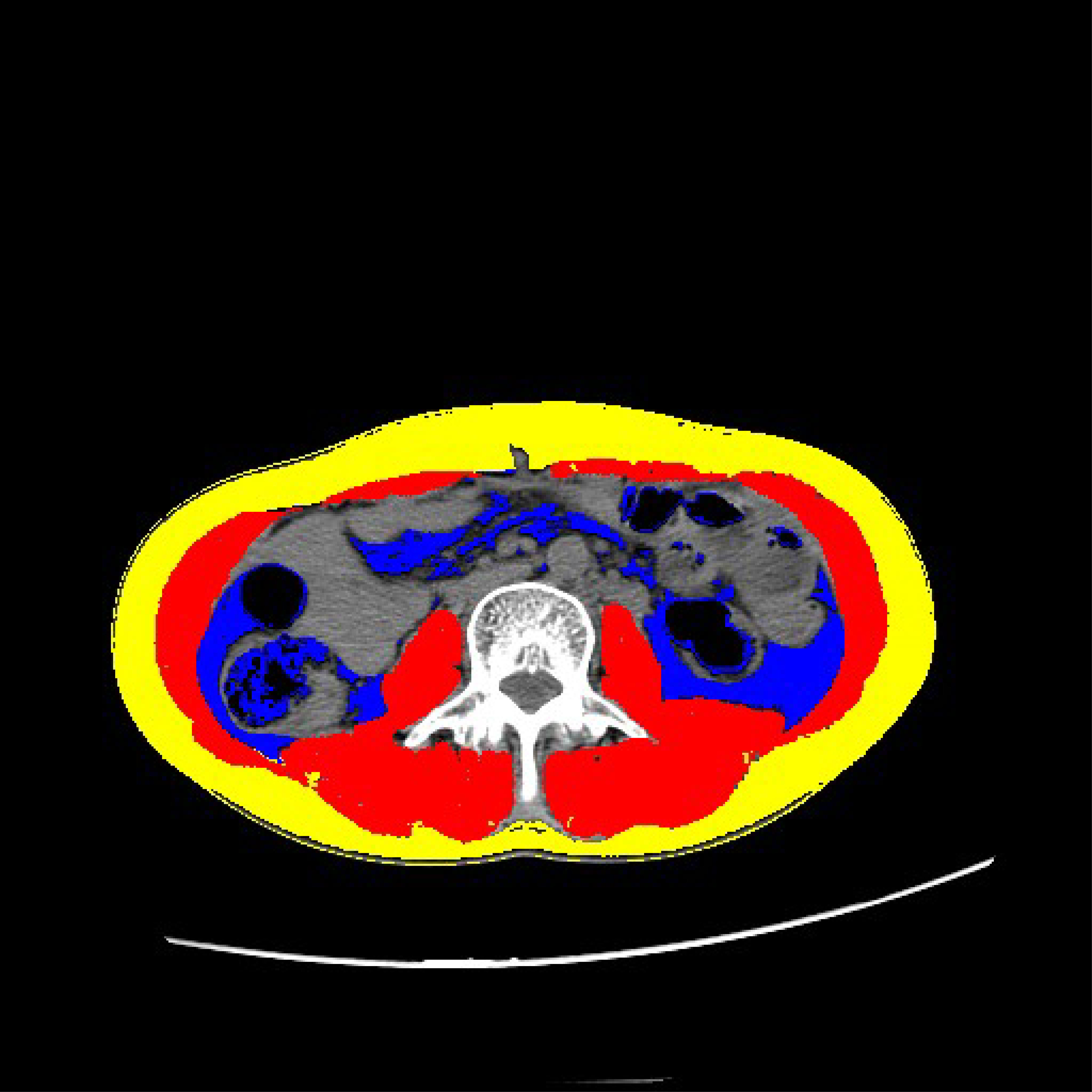

Supplement: Supplementary Figure 1 — The body composition analysis were segmented using standard Hounsfield unit (HU) and different color (skeletal muscle −29 to 150: red, SAT −190 to −30: yellow, and VAT −150 to −50: blue). [file Image_1.TIF]
